# Supplementary material for: New Titanium(IV)-Alkoxide Complexes Bearing Bidentate OO Ligand with the Camphyl Linker as Catalysts for High-Temperature Ethylene Polymerization and Ethylene/1-Octene Copolymerization
Source: Polymers (Basel). 2022 Nov 4;14(21):4735. doi: 10.3390/polym14214735 (PMC9658800; doi:10.3390/polym14214735)
Supplement: Supplementary file 1 [file polymers-14-04735-s001.zip › polymers-1927220-SM.pdf]

polymers

MDPI

Supporting information

New Titanium(IV) Alkoxide Complexes Bearing Bidentate OO Ligand with the Camphyl Linker as catalysts for High Temperature Ethylene Polymerization and Ethylene/1-Octene Copolymerization

Vladislav A. Tuskaev, Svetlana Ch. Gagieva, Dmitrii A. Kurmaev, Yulia V. Nelyubina, Petr V. Primakov, Maria D. Evseeva, Evgenii K. Golubev, Mikhail I. Buzin, Galina G. Nikiforova, Pavel B. Dzhevakov, Viktor I. Privalov, Kasim F. Magomedov and Boris M. Bulychev.

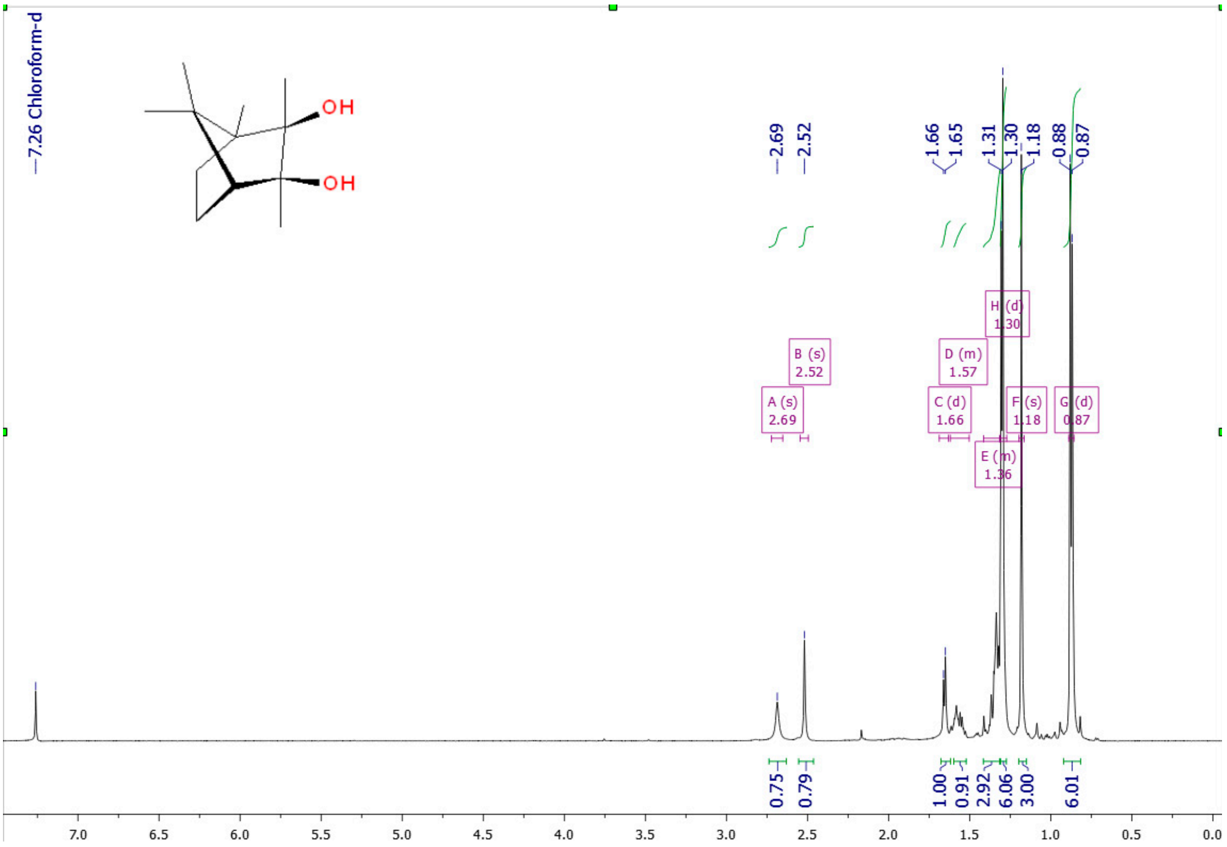

Figure S1. ^1H NMR spectrum of **L2** (400 MHz, $CDCl_3$).

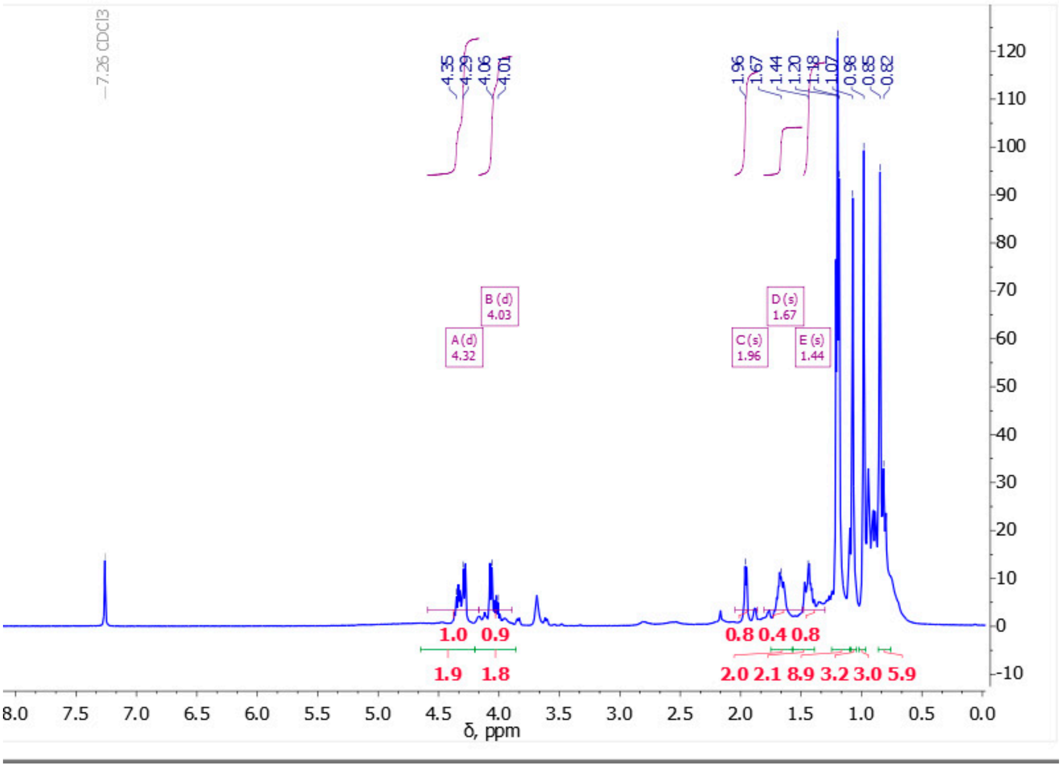

Figure S2. ^{1}H NMR spectrum of **[L¹Ti(OiPr)₂]₂** (400 MHz, $CDCl_3$).

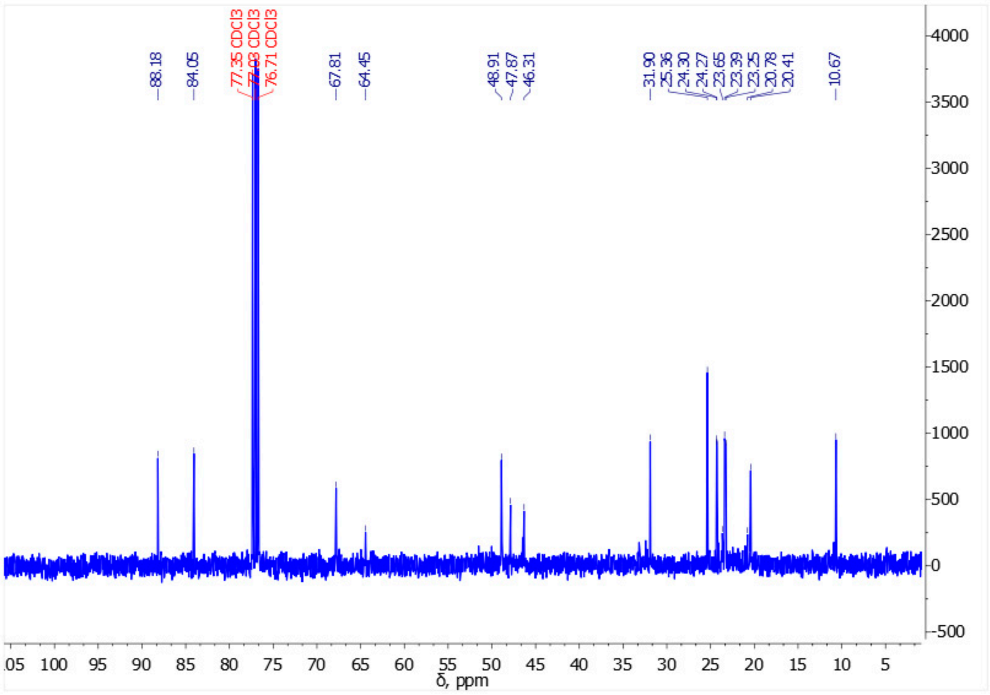

Figure S3. ^{13}C NMR spectrum of **[L¹Ti(OiPr)₂]₂** (400 MHz, $CDCl_3$).

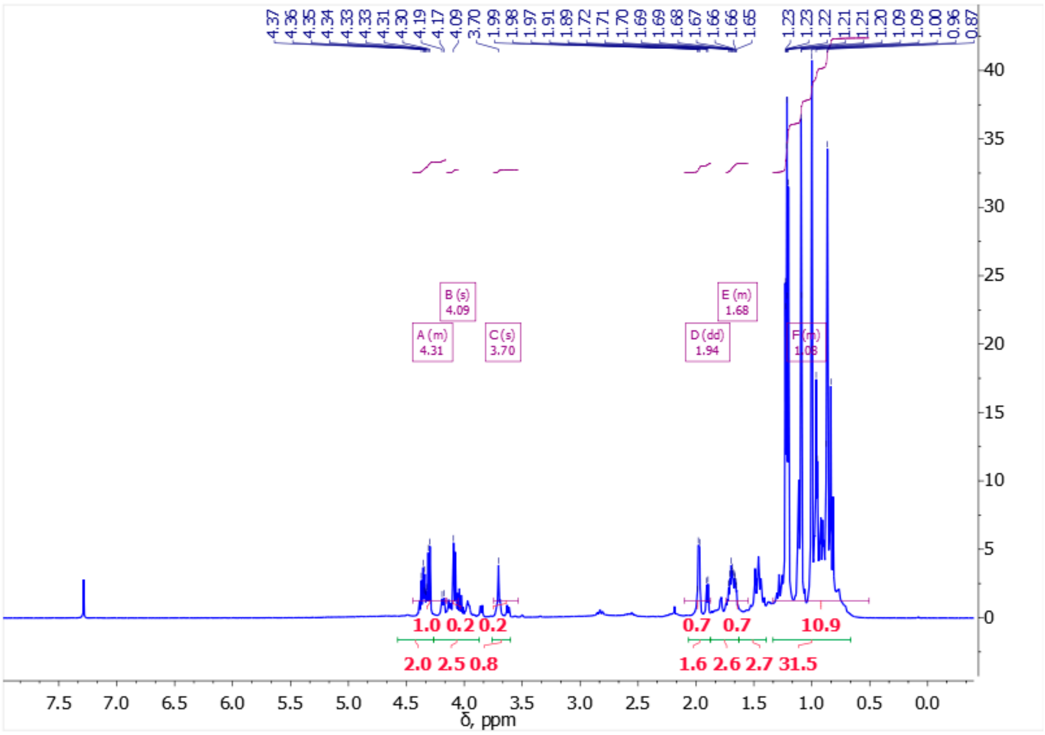

Figure S4. ^{1}H NMR spectrum of **[L[2]Ti(OiPr)2]2** (400 MHz, $CDCl_3$).

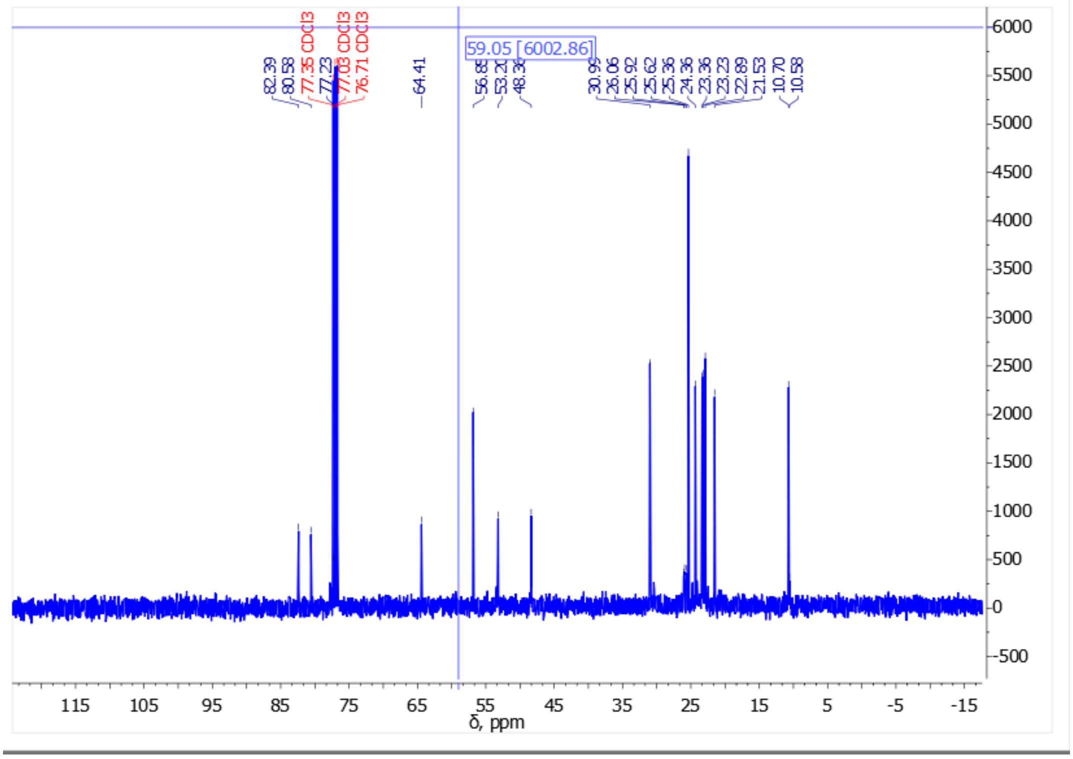

Figure S5. ^{13}C NMR spectrum of **[L[2]Ti(OiPr)2]2** (400 MHz, $CDCl_3$).

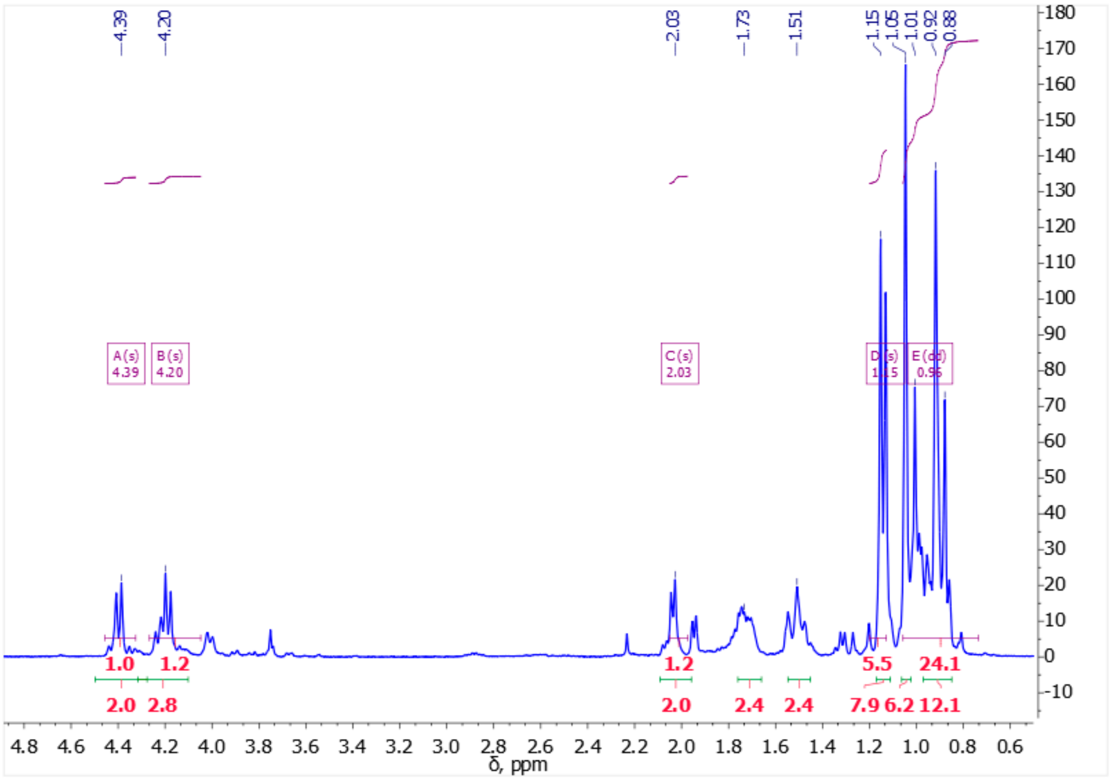

Figure S6. ^1H NMR spectrum of $\mathbf{L^1TiCl_2\ 2iPrOH}$ (400 MHz, $CDCl_3$).

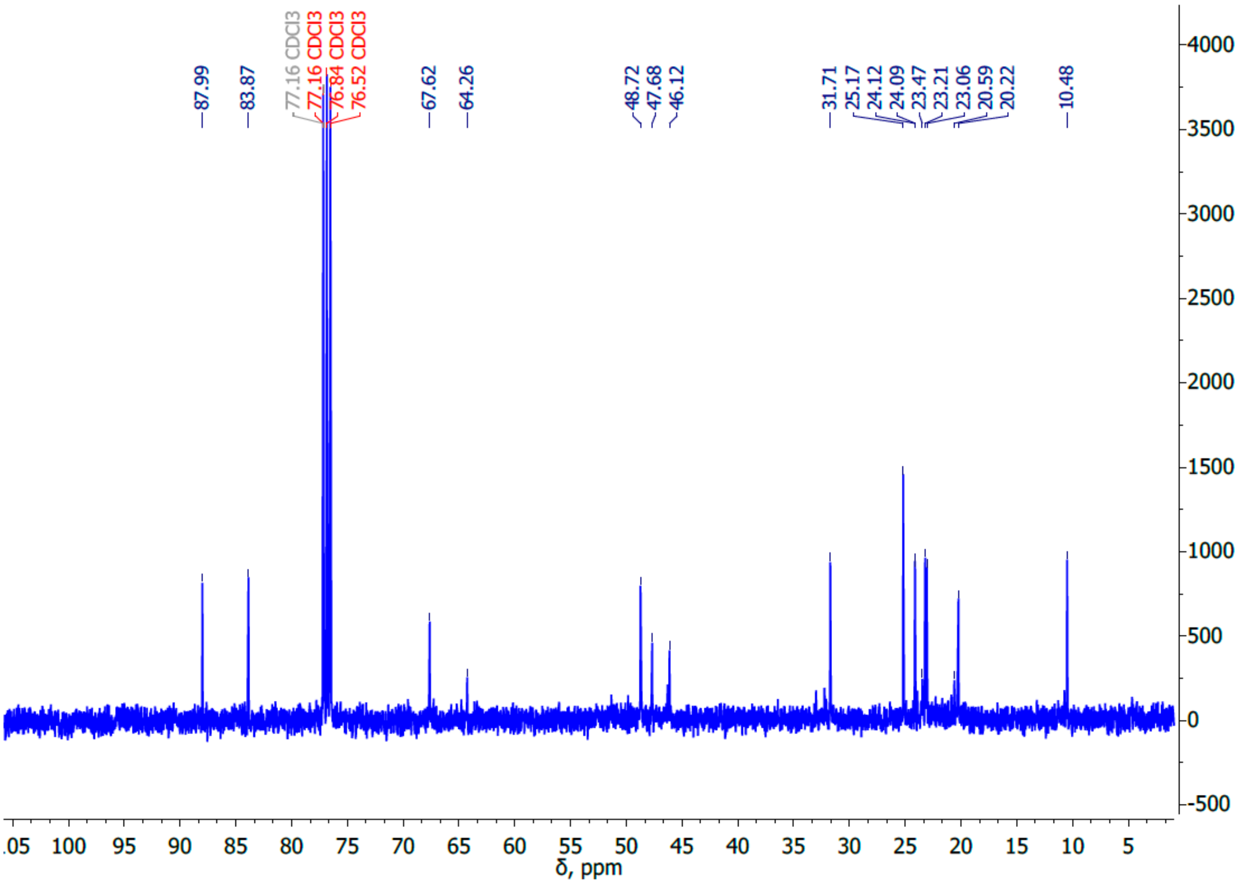

Figure S7. ^{13}C NMR spectrum of $\mathbf{L^1TiCl_2\ 2iPrOH}$ (400 MHz, $CDCl_3$).

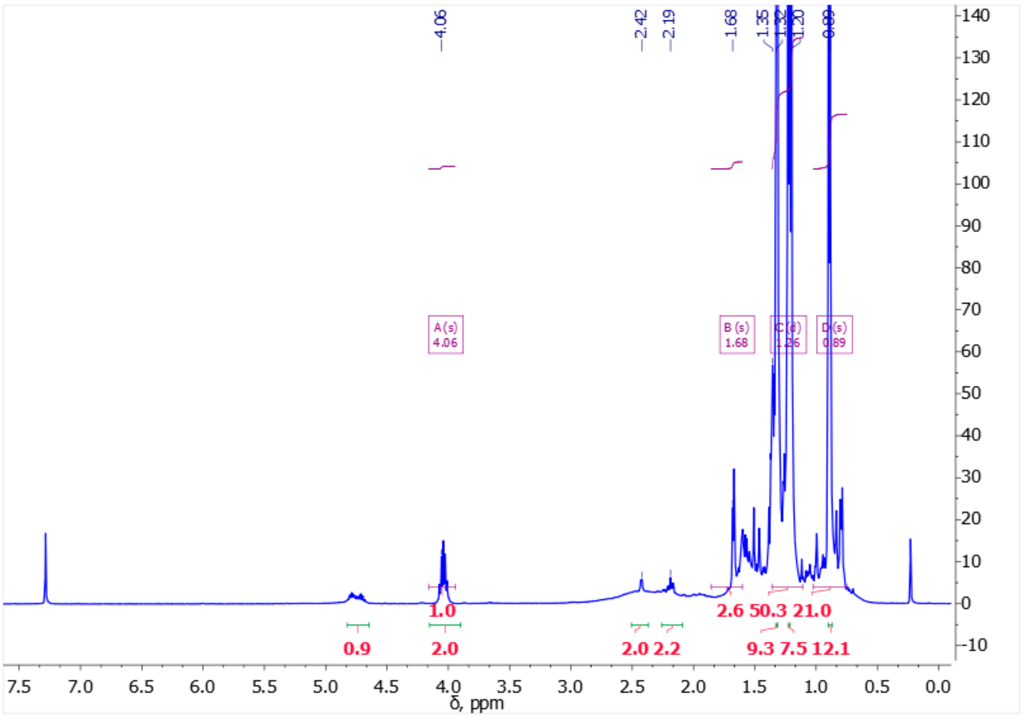

Figure S8. ^{1}H NMR spectrum of **L^2TiCl_2 2iPrOH** (400 MHz, $CDCl_3$).

Table S1. Crystal data and structure refinement parameters for **1** and **2**.

**Parameters**	**1**	**2**
Empirical formula	$C_{36}H_{68}O_8Ti_2$	$C_{32}H_{58}O_8Ti_2$
Formula weight	724.70	666.58
Crystal system	Triclinic	Triclinic
Space group	*P -1*	*P -1*
a (Å)	9.4637(4)	9.8892(4)
b (Å)	10.4537(4)	10.3689(5)
c (Å)	11.4316(4)	10.564(3)
α (°)	89.931(2)	62.165(3)
β (°)	77.573(2)	79.811(3)
γ(°)	85.344(2)	89.381(3)
V (Å^3)	1100.64(7)	939.5(2)
Z	1	1
D_{calc} (g cm^3)	1.093	1.178
μ(Mo-K$_\alpha$) (cm^{-1})	4.03	4.67
$2\theta_{max}$ (deg.)	52	52
F(000)	392	358
R_{int}	0.0334	0.0355
Collected reflections	12493	7873
Independent reflections	5312	3641
Observed reflections with $I > 2\sigma(I)$	4537	2670
Parameters	221	227
GooF	1.036	1.072
R_1, wR_2 ($I > 2\sigma(I)$)	0.0369, 0.1004	0.0616, 0.1600
$\Delta\rho_{max}$, ρ_{min} (e/Å^3)	0.358/-0.340	0.368/-0.331

Table S2. Selected geometric parameters and Continuous Symmetry Measures for **1** and **2**.[a]

	1	2
Ti-O(i-Pr), Å	1.7794(12), 1.7959(12)	1.775(11), 1.778(10)
Ti-O(L), Å	1.8396(10), 2.0038(10), 2.094(10)	1.842(3), 1.998(3), 2.0128(17)
Ti-O-Ti, °	108.94(5)	110.22(10)
Ti…Ti, Å	3.2822(4)	3.2097(15)
**PP-5**	29.048	26.961
**vOC-5**	3.036	3.523
**TBPY-5**	5.260	5.139
**SPY-5**	1.007	2.006

[a]L = Camphorquinone ligand; **PP-5** – Pentagon; **vOC-5** – Vacant octahedron; **TBPY-5 –** Trigonal bipyramid; **SPY-5** – Square pyramid.

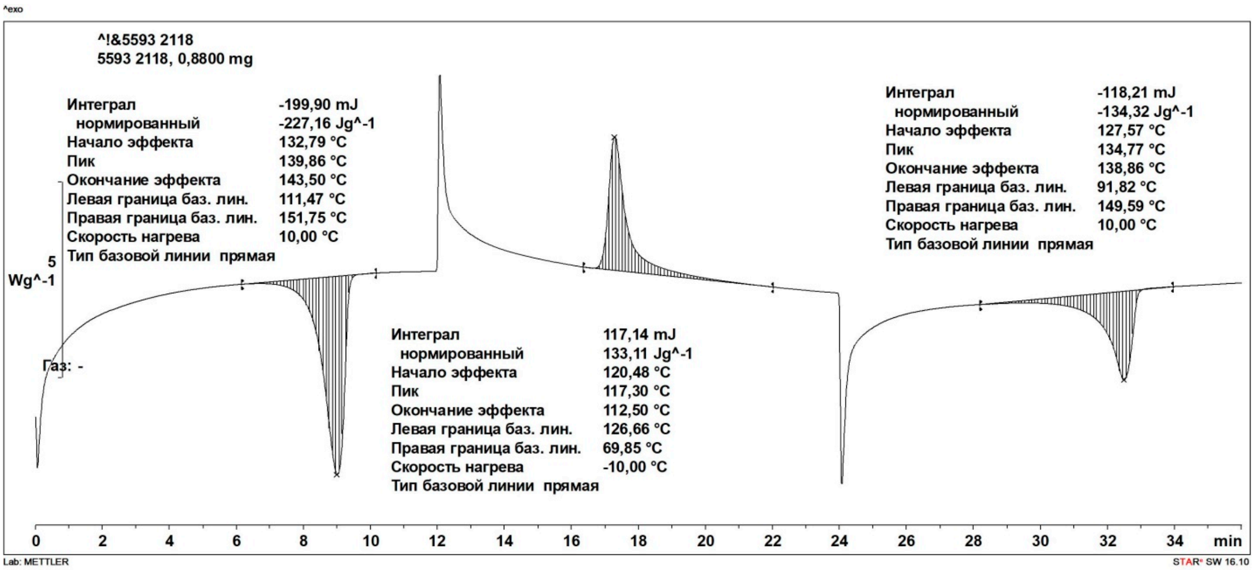

Figure S9. DSC curves corresponding to UHMWPE produced on **1**/Et_2AlCl+Bu_2Mg (entry 1, table 1).

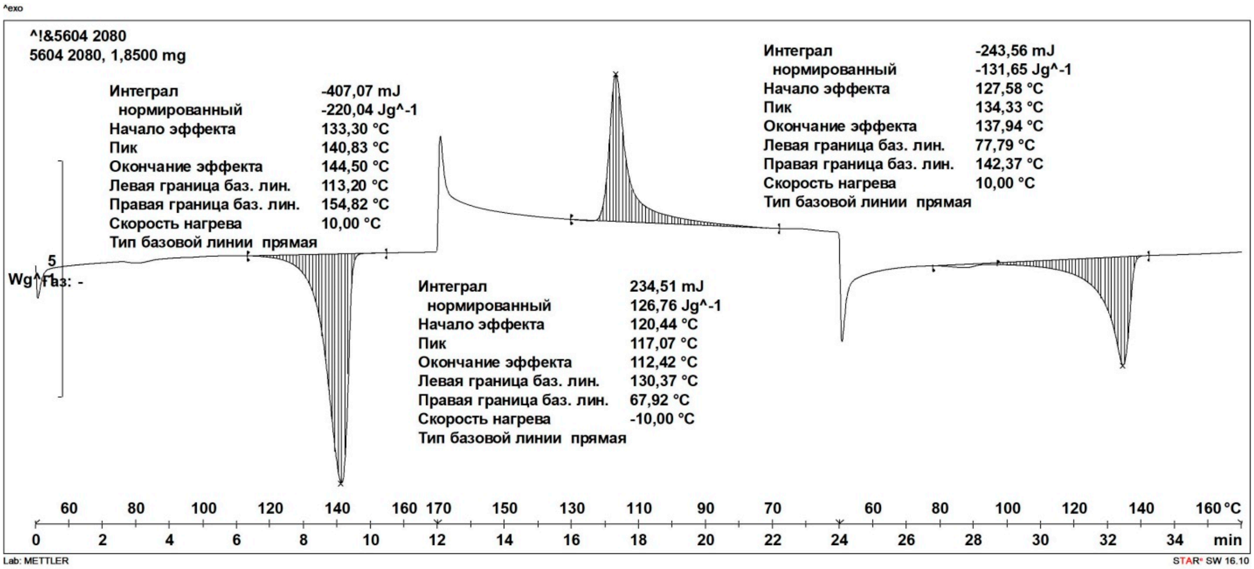

Figure S10. DSC curves corresponding to UHMWPE produced on **1**/$EtAlCl_2$+Bu_2Mg (entry 4, table 1).

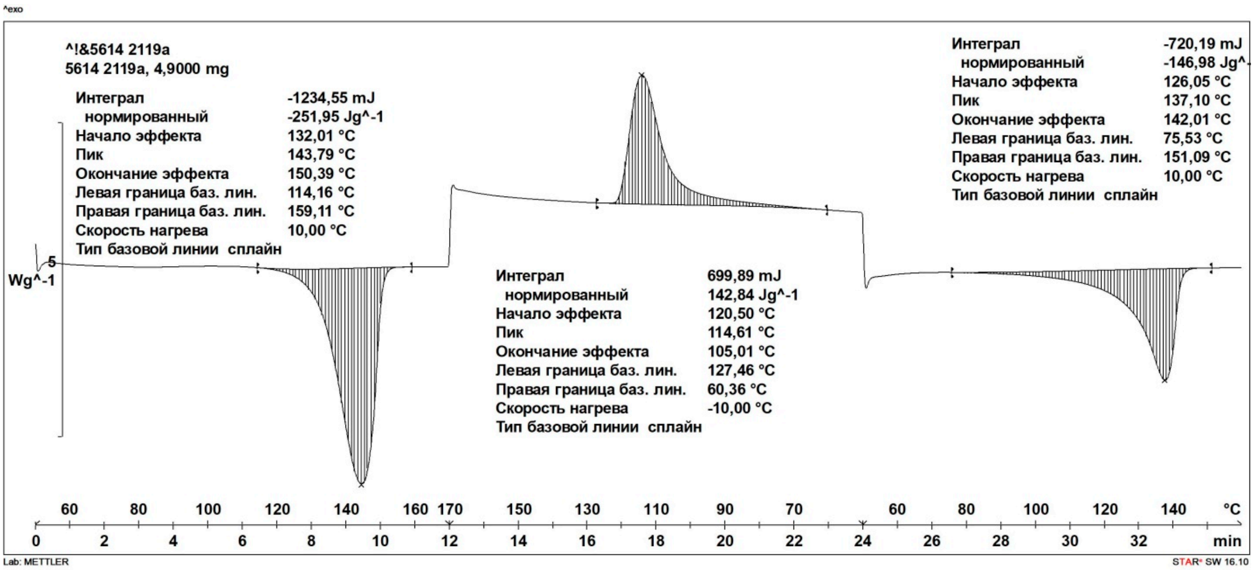

Figure S11. DSC curves corresponding to UHMWPE produced on **1**/ $Et_3Al_2Cl_3$+Bu_2Mg, nefras (entry 5, table 1).

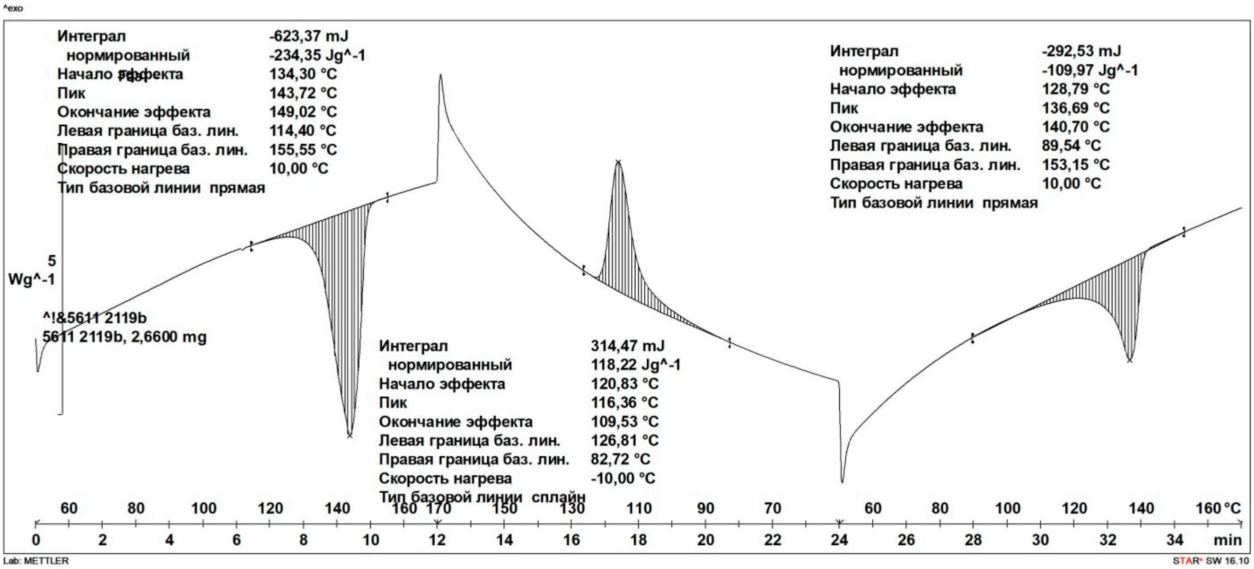

Figure S12. DSC curves corresponding to UHMWPE produced on **1**/ $Et_3Al_2Cl_3$+Bu_2Mg, nefras, pre-activation (entry 6, table 1).

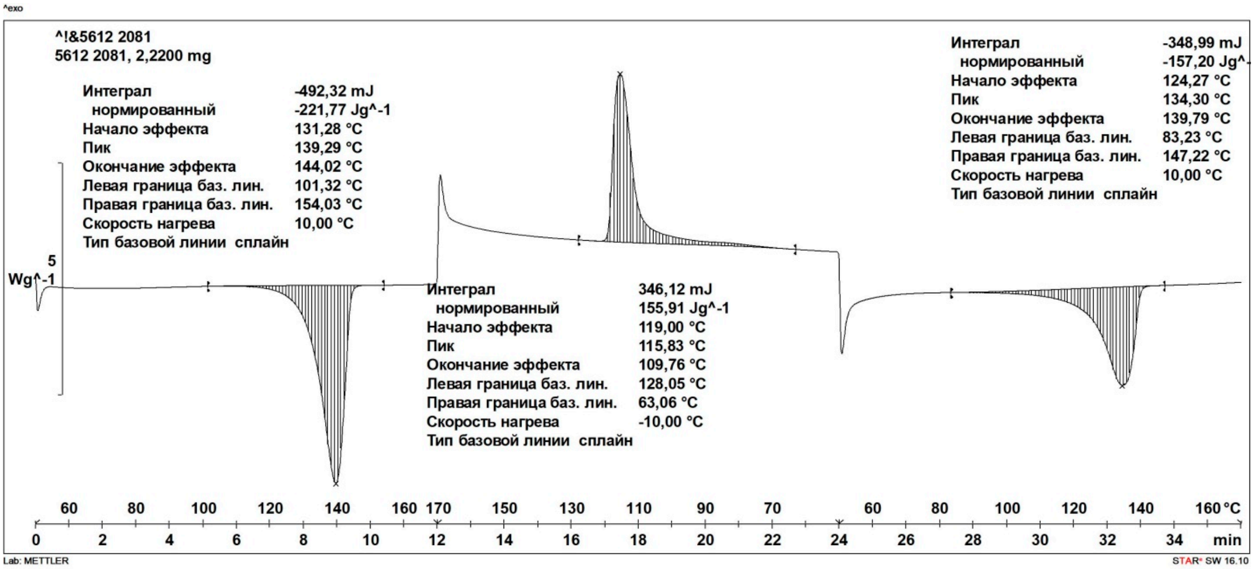

Figure S13. DSC curves corresponding to UHMWPE produced on **1**/ $Et_3Al_2Cl_3$+Bu_2Mg (entry 8, table 1).

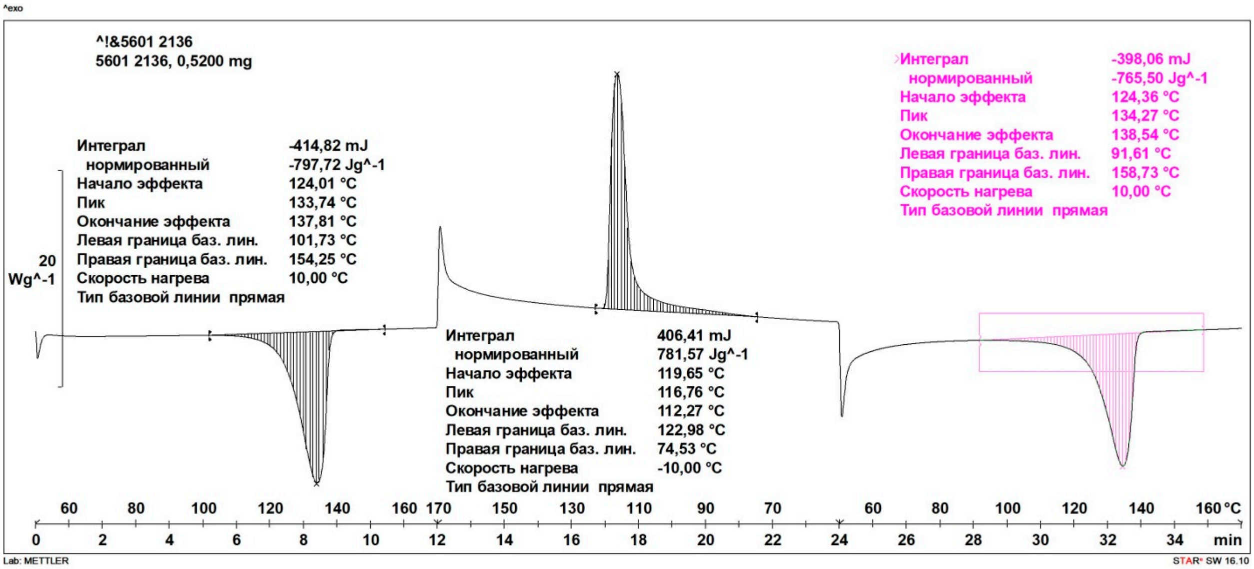

Figure S14. DSC curves corresponding to UHMWPE produced on **1**/$Et_3Al_2Cl_3$+Bu_2Mg, 70 °C (entry 9, table 1).

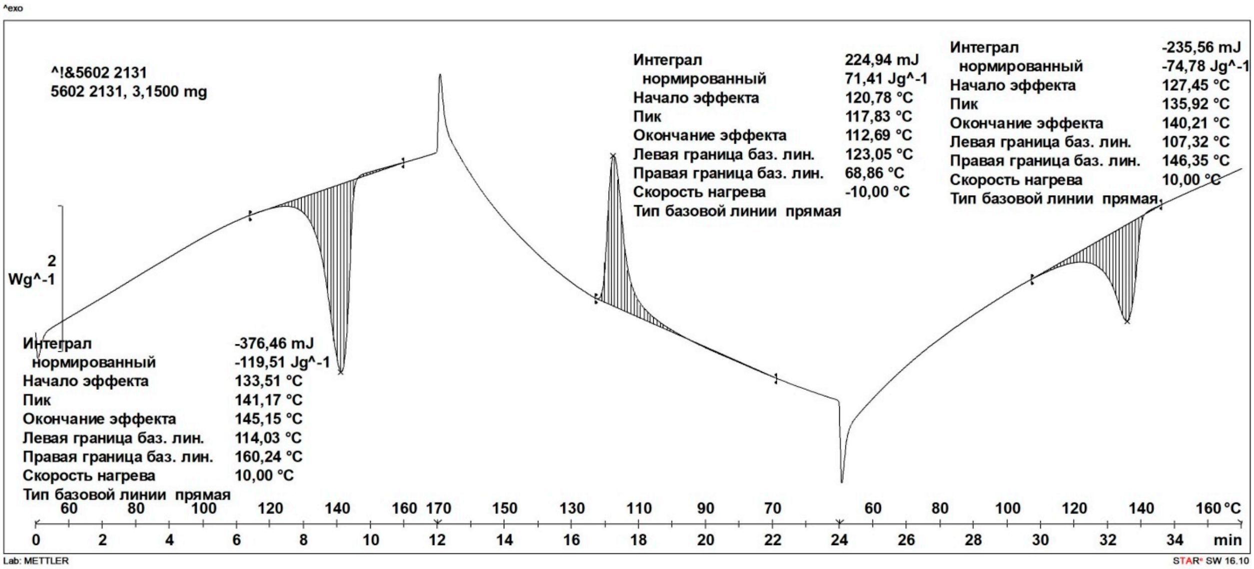

Figure S15. DSC curves corresponding to UHMWPE produced on **3**/ Et_2AlCl+Bu_2Mg (entry 15, table 1).

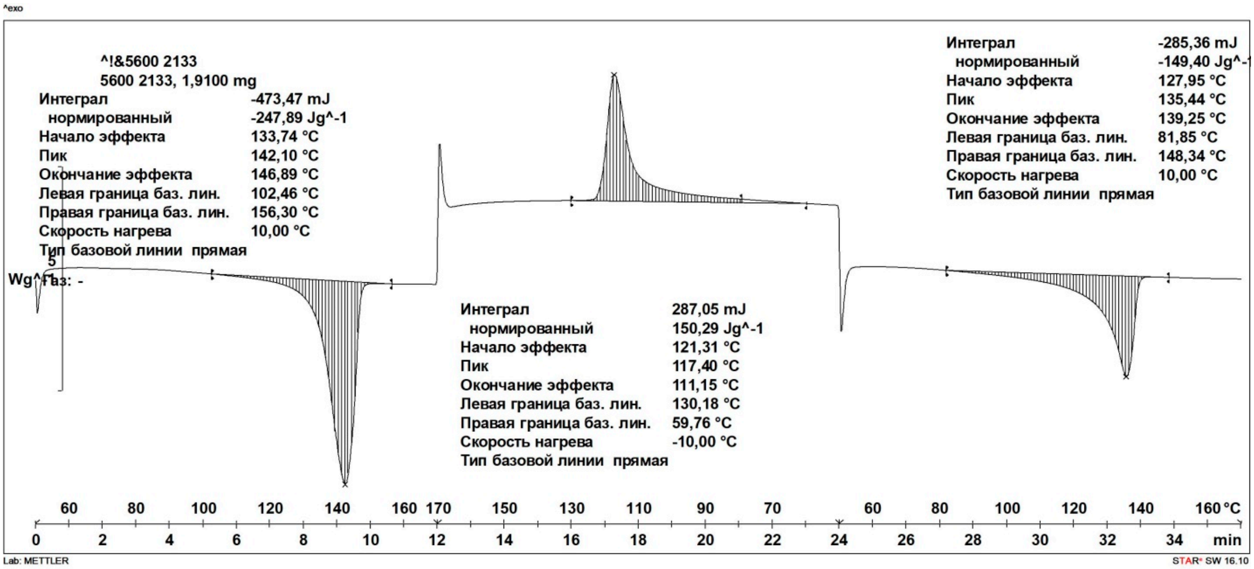

Figure S16. DSC curves corresponding to UHMWPE produced on **3**/ $EtAlCl_2$+Bu_2Mg (entry 17, table 1).

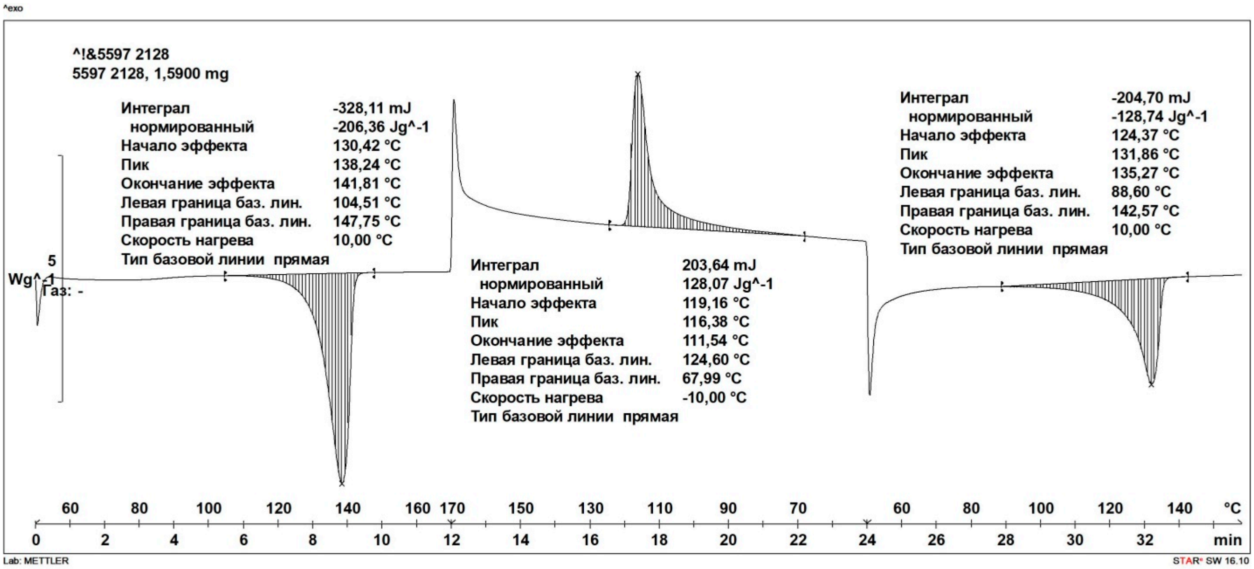

Figure S17. DSC curves corresponding to ethylene/1-octene copolymer produced on **1**/$Et_3Al_2Cl_3$+Bu_2Mg, 10°C (entry 1, table 3).

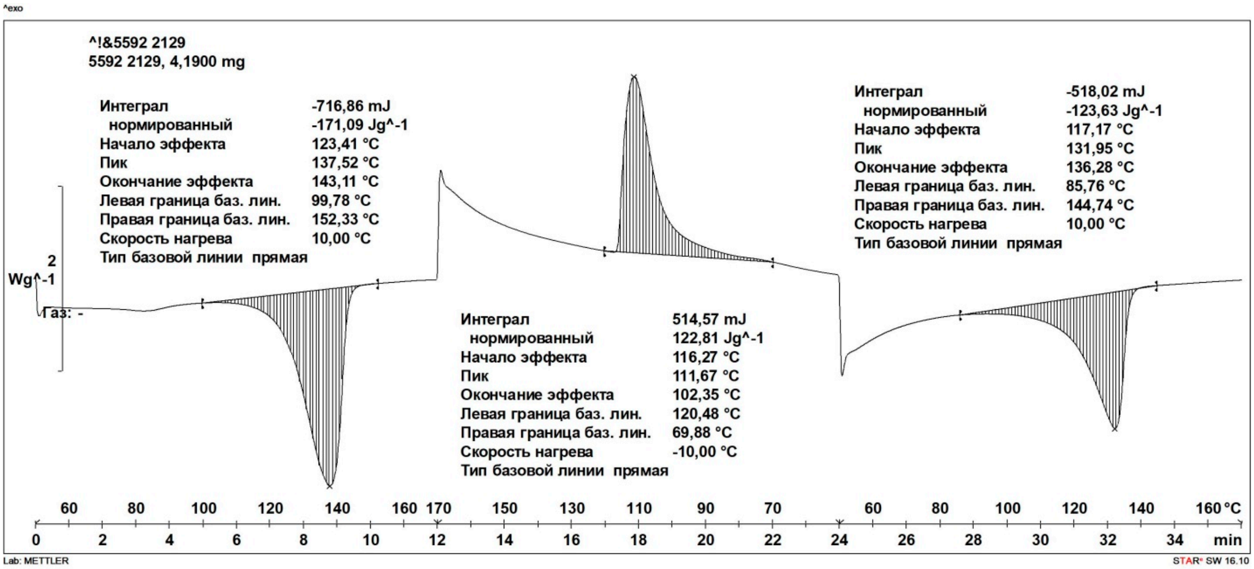

Figure S18. DSC curves corresponding to ethylene/1-octene copolymer produced on **1**/$Et_3Al_2Cl_3$+Bu_2Mg, 30°C (entry 2, table 3).

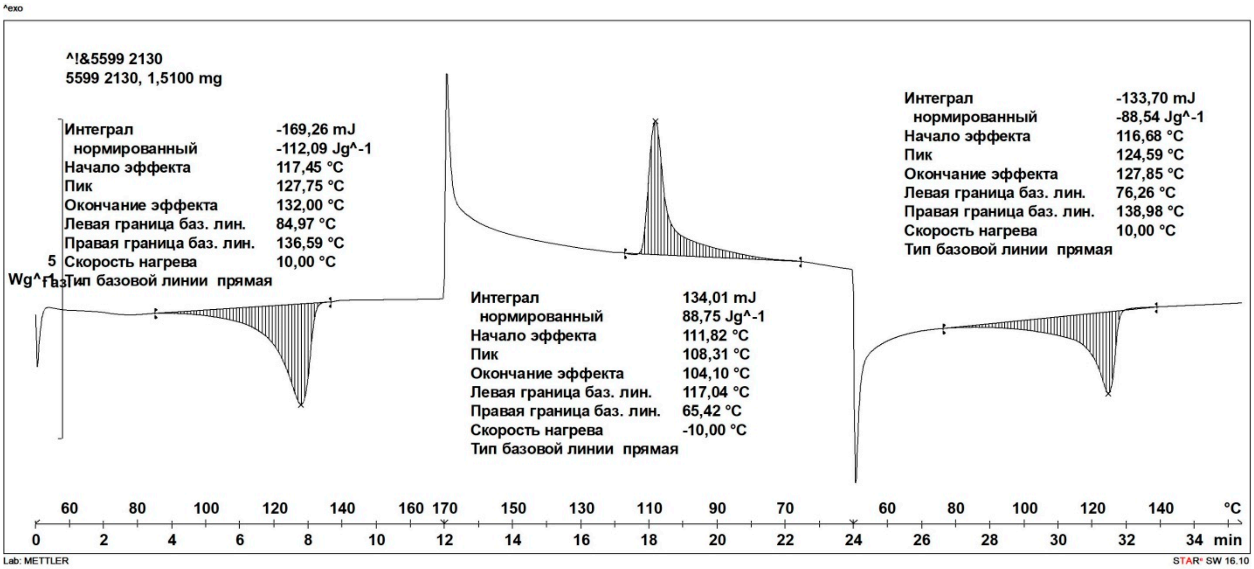

Figure S19. DSC curves corresponding to ethylene/1-octene copolymer produced on **1**/ $Et_3Al_2Cl_3$+Bu_2Mg, 50°C (entry 3, table 3).

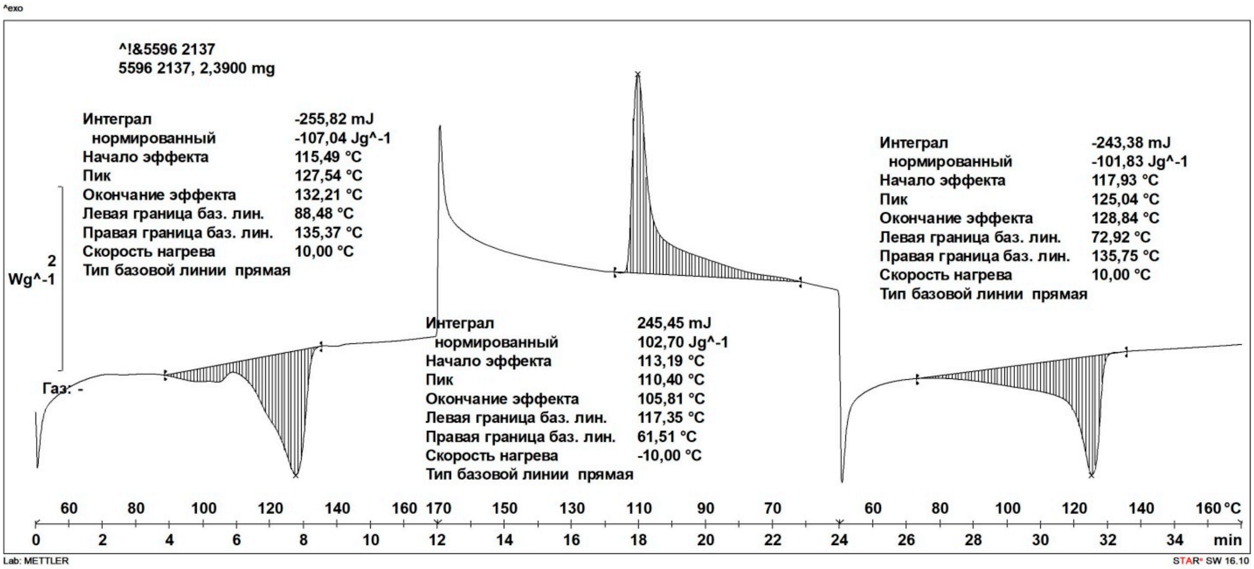

Figure S20. DSC curves corresponding to ethylene/1-octene copolymer produced on **1**/ $Et_3Al_2Cl_3$+Bu_2Mg, 70°C (entry 4, table 3).

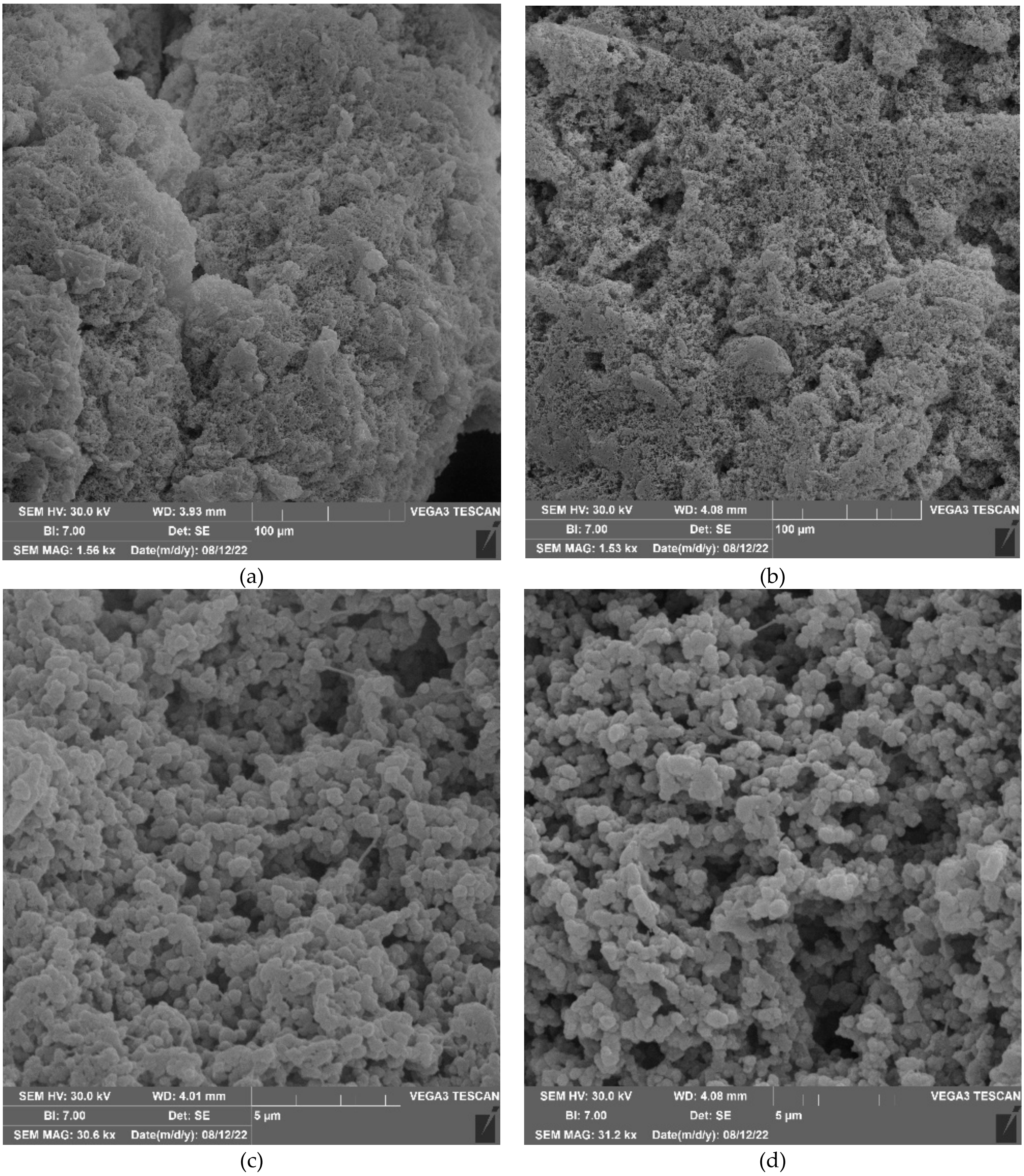

Figure 21. SEM images of the surface morphology of UHMWPE powders obtained with catalytic system **1**/$Et_3Al_2Cl_3$+Bu_2Mg, entry 8, 50 °C and **3**/ $Et_3Al_2Cl_3$+Bu_2Mg, entry 16, 50 °C .

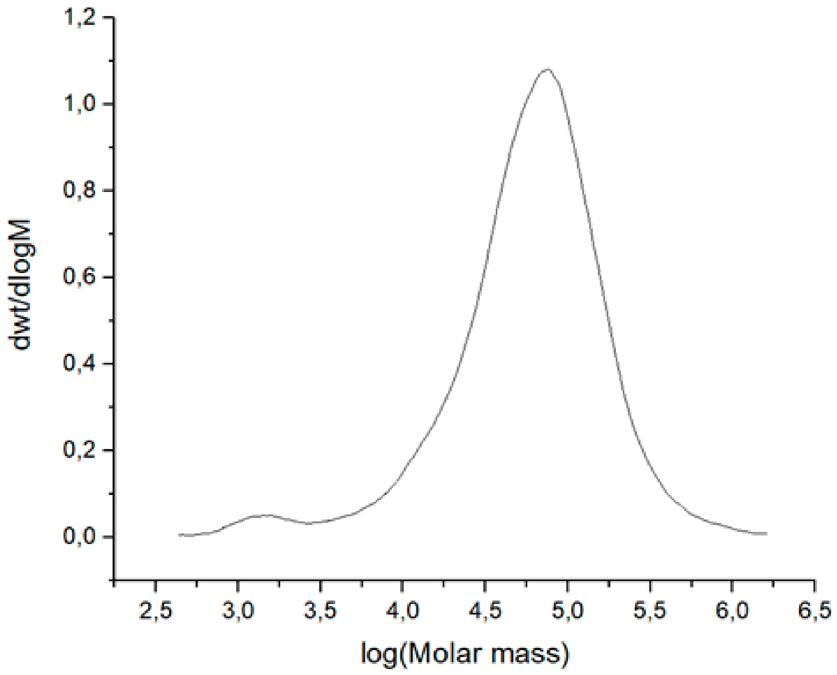

Figure S22. GPC curves corresponding to ethylene/1-octene copolymer produced on **1**/ $Et_3Al_2Cl_3$+Bu_2Mg, 10°C (entry 1, table 3).

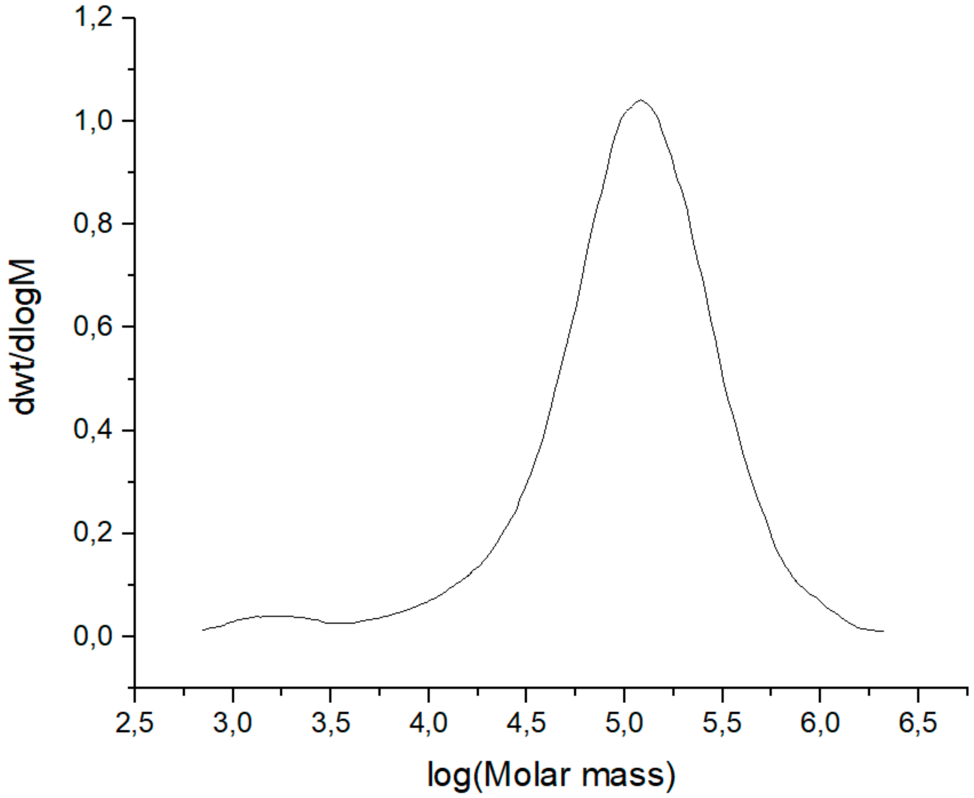

Figure S23. GPC curves corresponding to ethylene/1-octene copolymer produced on **1**/ $Et_3Al_2Cl_3$+Bu_2Mg, 30°C (entry 2, table 3).

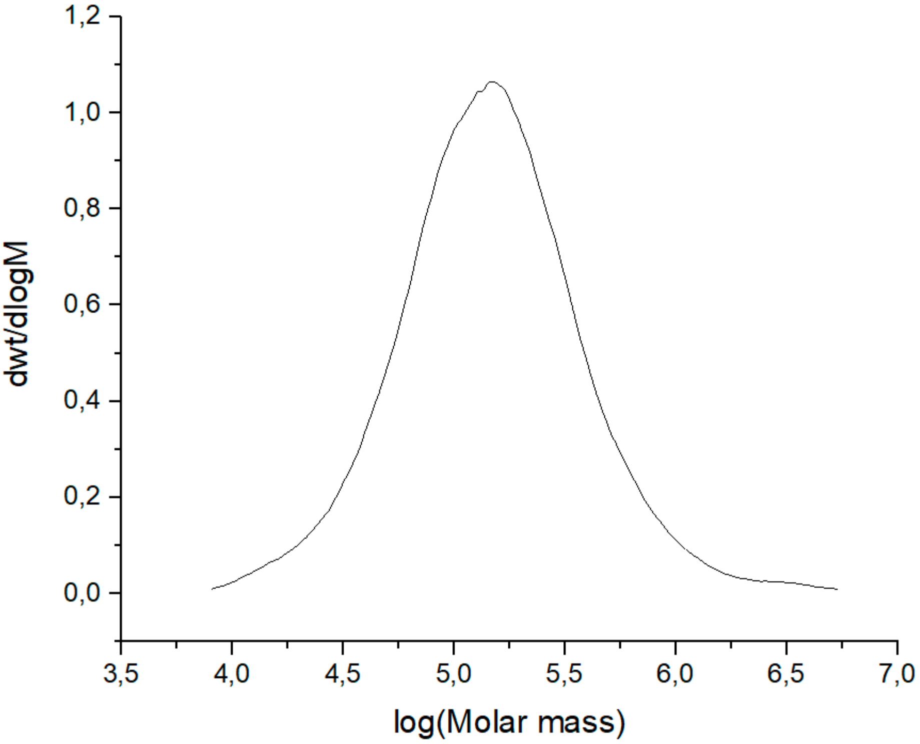

Figure S24. GPC curves corresponding to ethylene/1-octene copolymer produced on **1**/ $Et_3Al_2Cl_3$+Bu_2Mg, 50°C (entry 3, table 3).

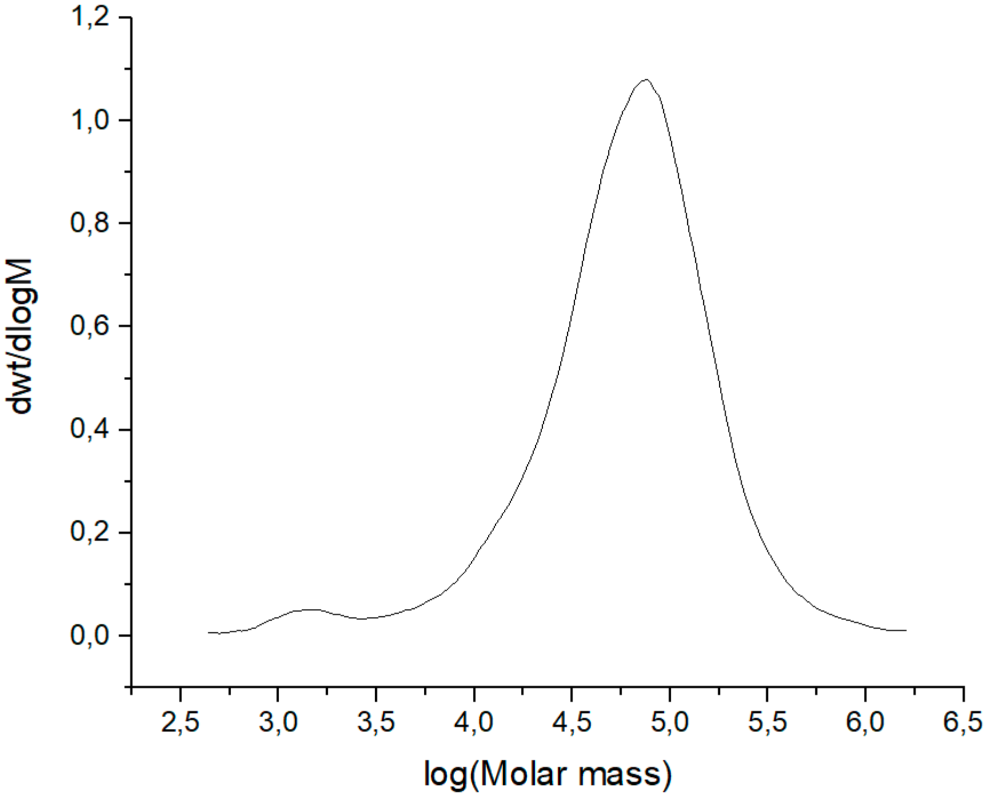

Figure S25. GPC curves corresponding to ethylene/1-octene copolymer produced on **1**/ $Et_3Al_2Cl_3$+Bu_2Mg, 70°C (entry 4, table 3).

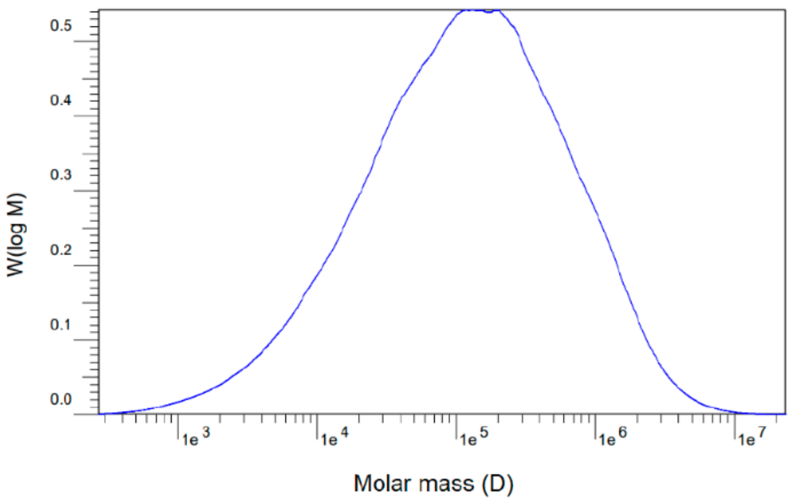

Figure S26. GPC curves corresponding to ethylene/1-octene copolymer produced on **2**/ $Et_3Al_2Cl_3$+Bu_2Mg, 10°C (entry 5, table 3).

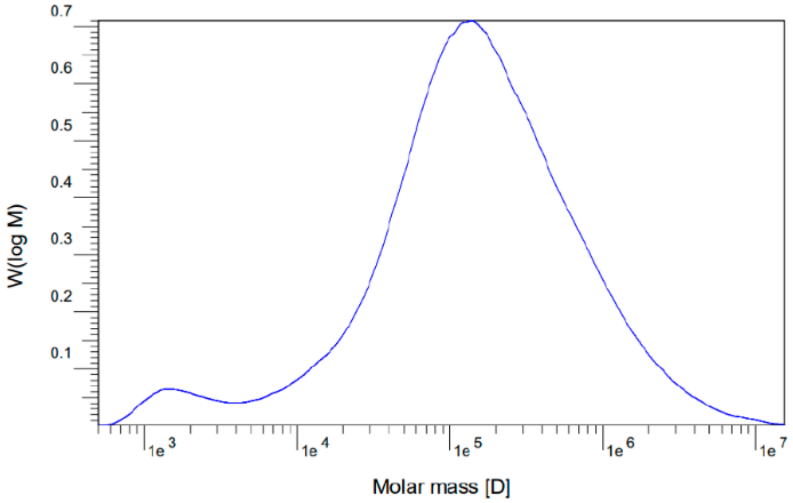

Figure S27. GPC curves corresponding to ethylene/1-octene copolymer produced on **2**/ $Et_3Al_2Cl_3$+Bu_2Mg, 30°C (entry 6, table 3).

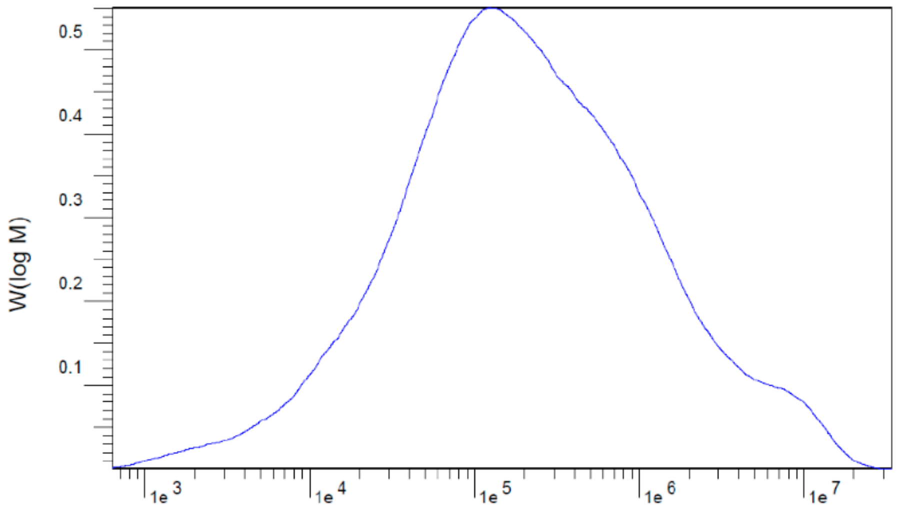

Figure S28. GPC curves corresponding to ethylene/1-octene copolymer produced on **2**/ $Et_3Al_2Cl_3$+Bu_2Mg, 50°C (entry 7, table 3).

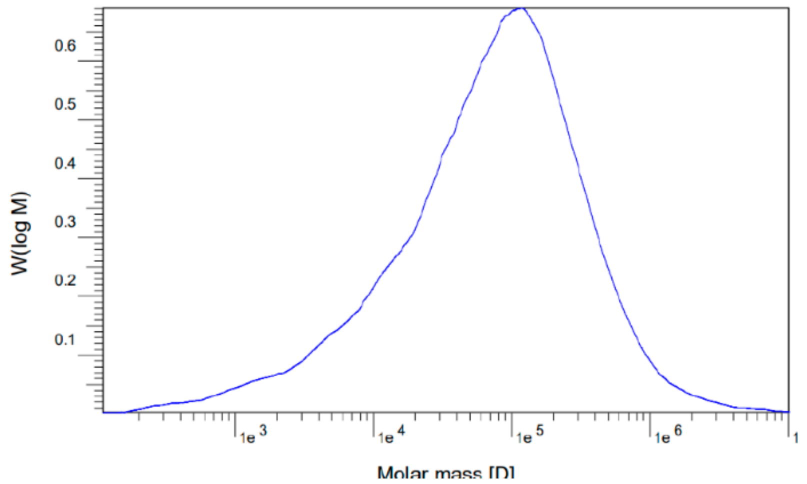

Figure S29. GPC curves corresponding to ethylene/1-octene copolymer produced on **2**/ $Et_3Al_2Cl_3$+Bu_2Mg, 70°C (entry 8, table 3).
